# Estimation of genetic parameters for pre-weaning growth traits in Dorper sheep under local Chinese conditions

**DOI:** 10.5713/ab.250570

**Published:** 2025-11-14

**Authors:** Ruijun Wang, Xinle Wang, Lifei Zhang, Yue Shi, Baodong Liu, Jing Li, Dayong Chen, Yunhui Ma, Huijie He, Jie Liu, Yongbin Liu, Yanjun Zhang

**Affiliations:** 1 College of Animal Science, Inner Mongolia Agricultural University, Hohhot, China; 2 Inner Mongolia Sino Sheep Technology Co., Ltd., Ulanqab, China; 3 Key Laboratory of Sheep Genetic Breeding and Reproduction Technology, Ministry of Agriculture and Rural Affairs, Ulanqab, China; 4 Key Laboratory of Mutton Sheep Genetics and Breeding, Ministry of Agriculture, Hohhot, China; 5 Inner Mongolia Key Laboratory of Sheep & Goat Genetics, Breeding and Reproduction, Hohhot, China

**Keywords:** Dorper Sheep, Genetic Correlation, Genetic Parameters, Phenotypic Correlation, Pre-weaning Growth Traits

## Abstract

**Objective:**

This study aimed to estimate non-genetic factors, variance components, and genetic parameters, including heritability, genetic/phenotypic correlations for birth weight (BW), weaning weight (WW), average daily gain (ADG), and Kleiber ratio (KR) traits of Dorper sheep under localized Chinese conditions.

**Methods:**

Data from 2,022 Dorper sheep lambs, collected between 2019 and 2021 at Inner Mongolia Sano Sheep Breeding Co., Ltd. were analyzed. Traits included BW, WW adjusted to 90 days, ADG, and KR. Generalized linear model (R 4.3.1) assessed non-genetic factors, including recipient dam age, sex, birth year, month, and herd. Six animal models were evaluated using ASReml’s AIREML to determine the most suitable model for estimating genetic parameters while bivariate models were utilized to analyze genetic and phenotypic correlations.

**Results:**

Recipient dam age, sex, birth year, month, and herd significantly affected all traits (p<0.05). Model 2, which incorporates direct additive genetic and maternal permanent environmental effects, was determined to be optimal. Heritability was low (BW: 0.0215; WW: 0.0287; ADG: 0.0391; KR: 0.0504). BW showed a negative genetic correlation with WW, ADG, and KR. In contrast, WW showed a strong positive genetic correlation with ADG (0.9952) and KR (0.9984), along with high phenotypic correlations with these traits (0.9829 and 0.8819, respectively).

**Conclusion:**

The low heritability limits direct selection for pre-weaning traits. Prioritizing WW enhances indirect genetic gains for ADG and KR, facilitating the optimization of Dorper sheep breeding strategies under Chinese intensive systems.

## INTRODUCTION

Sheep are a globally vital livestock resource and hold strategic importance for China’s animal husbandry. China possesses 7.7% of global sheep genetic resources, with 110 sheep breeds (including 58 native breeds, 38 cultivated breeds, and 14 introduced breeds), forming a genetically diverse germplasm system optimized for meat, wool and fur production [1,2]. In 2023, sheep stock reached 192.98 million, with mutton production at 5.18 million tons [3]. However, the mutton market still faces an annual supply-demand gap of 416,000 tons due to rising consumer [4], highlighting the urgency of accelerating high-quality breeding. Breed improvement has advanced via targeted introduction global germplasm like Dorper sheep, leveraging hybrid vigor to develop climate-resilient breeds adapted to northern China’s semi-arid regions [5]. Dorper sheep, a South African meat sheep breed, have gained global prominence for their adaptability and productivity [6]. This breed was developed in the 1930s by crossing Blackhead Persian sheep (dam line) and Dorset Horn sheep (sire line) [7]. Since China introduced Dorper sheep from Australia in 2001, the breed has been expanded and popularized it in Henan, Anhui, Inner Mongolia, Jilin, and other places through embryo transfer and other techniques. The Dorper sheep have been used as the terminal parent for crossbreeding with local breeds, giving rise to newly cultivated breeds such as Huanghuai meat sheep [8], Dumeng sheep [4], Luxi blackhead sheep [9], and Shuangqian meat sheep [10], which exhibit pronounced hybrid vigor.

Pre-weaning traits (birth weight [BW], weaning weight [WW], average daily weight gain [ADG] and Kleiber ratio [KR]) directly determine production efficiency in large-scale breeding. Genetic parameter estimation is essential for breeding strategy optimization. It reported estimates of heritability of WW include 0.23 in Akkaraman sheep [11], 0.22 in Dağlıç Sheep [12], 0.31 in Doyogena sheep [13], 0.20 in Sangsari sheep [14]. Differences in the heritability of different breeds of sheep are influenced not only by genetic factors but also by environmental factors [15,16], necessitating a deep dissection of these components when estimating genetic parameters. Current Dorper sheep genetic evaluations focus on foreign populations, lacking systematic assessments of pre-weaning trait heritability, genetic correlations, and genotype-environment interactions in China. This study aimed to estimate variance components and genetic parameters for pre-weaning traits in Dorper sheep. By comparing and analyzing estimation discrepancies across different animal models, we determined the optimal model for early growth traits and study models’ feasibility in Dorper sheep under local Chinese conditions.

## MATERIALS AND METHODS

### Animals and data collection

All experimental data and pedigree records were obtained from Inner Mongolia Sano Sheep Breeding, including 2,022 early growth trait records of Dorper sheep collected from 2019 to 2021. The dataset was rigorously preprocessed in Excel: records with issues like incorrect ear tags, missing gender or production performance data, incomplete phenotypic measurements, and outliers outside “mean±3σ” were excluded, resulting in 1,999 lambing records involving 14 sires and 328 dams. The study focused on four early growth traits—BW, WW (adjusted to 90 days due to varying weaning ages), pre-weaning ADG, and pre-weaning KR. The KR is an indirect selection method used to measure feed conversion. The KR before weaning is calculated as follows [17]:


(1) $$ADG=\frac{W1-BW}{D}$$

(2) WW=BW+ADG×90

(3) $$KR=\frac{ADG}{WW^{0.75}}$$

Where, W1 = WW, D = number of days between weaning date and date of birth.

### Feeding practices

The experimental population was housed at the company’s production base situated in Ulanqab (41°10′−43°22′N, 110°20′−113°00′E), a semi-arid continental monsoon climate zone with 313.8 mm annual precipitation (60%–70% in summer) and summer temperatures averaging below 20°C.

The sheep were reproduced by artificial insemination and LOPU-IVF-ET at 18 months of age. Newborn lambs were ear-tagged and weighed within 24 hours post-partum, with dam-suckling permitted during grazing until 30 days old. From 2 months postpartum, lambs received twice-daily suckling sessions (morning/evening) alongside daytime hay supplementation until weaning. Post-weaning lambs were transitioned to unified feeding regimens in grouped pens.

### Fixed effects

The fixed factors, including the birth year (2019–2021), birth month (ten months), herd (ten herds), sex (male or female), and age of recipient dam (2–4 years old), were analyzed using a General Linear Model (GLM) procedure using R software ver. 4.3.1. Post-hoc pairwise comparisons were conducted using Duncan’s multiple range test to determine homogeneous subsets among significantly different factor levels. A statistical model of GLM for the trait of per-weaning is described below:


(4) $$y_{ijklmr}=\mu +Y_{i}+H_{j}+M_{k}+S_{l}+A_{m}+e_{ijklmr}$$

Where *y_ijklmr_* is the individual observation, μ is the overall mean, *Y_i_* is fixed effect of birth year, *H_j_* is fixed effect of herd, *M_k_* is fixed of birth month, *S_l_* is fixed of sex, and *A_m_* is fixed of age of recipient dam, and *e_ijklmr_* is the residual error.

### Genetic parameters estimation

Genetic parameters of pre-weaning growth traits were estimated by the AI-REML method using the ASReml ver. 4.1 [18]. Maternal permanent environmental effects (Model 2) were included to account for consistent non-genetic influences from recipient dams (e.g., milk quality, rearing behavior) across multiple offspring, a critical factor in embryo-transferred populations. Finally, we calculated the fitting parameters for six single-trait animal models and determined the optimal model for each trait using Akaike information criterion (AIC), Bayesian information criterion (BIC), and likelihood ratio test (LRT) tests. We estimated heritability using single-trait models, calculated genetic correlations between trait combinations using bivariate analysis, and then estimated heritability. The animal models are as follows:


(5) $$\text{Model }1:y=Xb+Z_{1}a+e$$

(6) $$\text{Model }2:y=Xb+Z_{1}a+Z_{3}c+e$$

(7) $$\text{Model }3:y=Xb+Z_{1}a+Z_{2}m+e\;with\;COV(\text{a},\text{m})=0$$

(8) $$\text{Model }4:y=Xb+Z_{1}a+Z_{2}m+e\;with\;COV(\text{a},\text{m})=A\sigma_{am}$$

(9) $$\text{Model }5:y=Xb+Z_{1}a+Z_{2}m+Z_{3}c+e\;with\;COV(\text{a},\text{m})=0$$

(10) $$y=Xb+Z_{1}a+Z_{2}m+Z_{3}c+e\;with\;COV(\text{a},\text{m})=A\sigma_{am}$$

Where *y* is a vector of observed traits; b, $a˜N(0,\;A\sigma_{a}^{2}),m˜N(0,\;A\sigma_{m}^{2}),\;\text{c}˜N(0,\;I_{d}\sigma_{a}^{2})$, are vectors of fixed, direct additive genetic, maternal additive genetic, maternal permanent environmental effects, respectively. $e˜N(0,\;I_{n}\sigma_{e}^{2})$ is a vector of residual effect; *X*, *Z*_1_, *Z*_2_, and *Z*_3_ are correlation structure matrices for these effects relating *b*, *a*, *m*, and *c* to *y*. It was assumed as follows [19]:


(11) $$\left[\begin{array}{cccc} A\sigma_{a}^{2} & A\sigma_{am} & 0 & 0\\ A\sigma_{ma} & A\sigma_{m}^{2} & 0 & 0\\ 0 & 0 & I_{d}\sigma_{c}^{2} & 0\\ 0 & 0 & 0 & I_{n}\sigma_{e}^{2} \end{array}\right]$$

*A* represent the additive genetic correlation matrix, and *I_d_* and *I_n_* are identity matrices of records equal to the number of dams and lambs, respectively. All components with the phenotypic variance ( $\sigma_{p}^{2}$) being the sum of $\sigma_{a}^{2},\sigma_{m}^{2},\sigma_{c}^{2}$, and $\sigma_{e}^{2}$ were derived at convergence.

The direct heritability:


(12) $$h_{d}^{2}=\frac{\sigma_{a}^{2}}{\sigma_{p}^{2}}$$

The maternal heritability:


(13) $$h_{m}^{2}=\frac{\sigma_{m}^{2}}{\sigma_{p}^{2}}$$

The proportion of phenotypic variance attributable to maternal permanent environmental effects:


(14) $$c^{2}=\frac{\sigma_{c}^{2}}{\sigma_{p}^{2}}$$

The total heritability:


(15) $$h_{t}^{2}=\frac{\sigma_{a}^{2}+\sigma_{m}^{2}+2\sigma_{am}}{\sigma_{p}^{2}}$$

The genetic correlation between the direct and maternal effects:


(16) $$r_{am}=\frac{\sigma_{am}}{\sqrt{\sigma_{a}^{2}\times \sigma_{m}^{2}}}$$

The direct-maternal correlation (*r_am_*) was computed as the ratio of the estimates of the direct maternal covariance (*σ_am_*) to the product of the square roots of the estimates of $\sigma_{a}^{2}$ and $\sigma_{m}^{2}$. The accuracy of the different model estimates was tested using the AIC as follow:


(17) AIC=2k-2In (L)

Where *L* represents the maximum likelihood value of the model, and k denotes the total number of parameters to be estimated.

A lower AIC value indicates a better balance between model fit and complexity, reflecting superior explanatory power while controlling for overfitting [20].

The BIC is a model selection criterion similar to AIC, balancing goodness-of-fit and complexity. The BIC was as follow:


(18) BIC=kln (n)-2ln (L)

Where *L* is the maximum likelihood value of the model, *k* is the number of parameters in the model, and n is the sample size. Consequently, the model with the smallest BIC among candidate models is typically selected as optimal.

To identify the most appropriate model for the target traits, a LRT was employed. This test evaluates the statistical significance of additional parameters by comparing the likelihood functions of nested models, ultimately determining the simplest model that retains strong explanatory power. A LRT was performed to obtain the appropriate model as given below [21]:


(19) $$LRT=-2\;\text{log}\frac{L_{1}}{L_{2}}$$

Where *L*_1_ and *L*_2_ denote the maximum likelihood values of model 1 and model 2, respectively. Model 1 is a sub-model of model 2. LR follows a chi-square distribution, with degrees of freedom equal to the difference in the number of parameters between Model 2 and Model 1. A statistically significant test result (p<0.05) indicates that the additional parameter(s) in Model 2 have a meaningful impact on the trait; otherwise, their effect is deemed non-significant. Furthermore, genetic and phenotypic correlations between traits were estimated using bivariate animal models. These models were constructed based on the optimal model selected for each trait. The structure of the bivariate model is as follows [22]:


(20) $$\left[\begin{array}{c} y_{1}\\ y_{2} \end{array}\right]=\left[\begin{array}{cc} X_{1} & 0\\ 0 & X_{2} \end{array}\right]\left[\begin{array}{c} b_{1}\\ b_{2} \end{array}\right]+\left[\begin{array}{cc} Z_{1} & 0\\ 0 & Z_{2} \end{array}\right]\left[\begin{array}{c} a_{1}\\ a_{2} \end{array}\right]+\left[\begin{array}{c} e_{1}\\ e_{2} \end{array}\right]$$

Where *y*_1_ and *y*_2_ represent the vectors of observed traits 1 and 2. *b*_1_ and *b*_2_ are the vectors of fixed effects. *a*_1_ and *a*_2_ denote the vectors of additive genetic effects. *e*_1_ and *e*_2_ correspond the vectors of residual effects. The design matrices *X*_1_ and *X*_2_ associate observations with fixed effects, while *Z*_1_ and *Z*_2_ link the traits to their respective additive genetic effects. Genetic and phenotypic correlations between the two traits were estimated following the methodology outlined by Alam et al [23].

## RESULTS

### Descriptive statistics

The description statistics for early growth traits are shown in Table 1. The average BW was 3.94 kg with a coefficient of variation (CV) of 18.38%. The average WW was 29.12 kg with a CV of 15.14%. The average ADG was 0.28 kg/d with a CV of 17.44%. The average KR was 0.02 with a CV of 6.69%. These values highlight significant trait variability, which is essential for the genetic assessment and improvement of Dorper sheep.

### Environmental effects of pre-weaning traits

The least square mean and standard deviation for each trait of Dorper sheep are shown in Table 2. The effects of recipient dam age, sex, birth year, birth month and herd were highly significant (p<0.01) on early growth traits. In terms of recipient dam age, lambs born to 2-year-old and 4-year-old dams exhibited significantly higher BW than those born to 3-year-old dams (p<0.05). Conversely, offspring of 3-year-old dams achieved the highest WW, ADG, and KR, which were significantly greater than those of lambs from 2-year-old and 4-year-old dams (p<0.05). With respect to sex, male lambs outperformed females across all growth traits, with statistically significant differences observed for each trait (p<0.05). Regarding birth year effects, lambs born in 2019 and 2021 had significantly higher BW compared to those born in 2020 (p<0.05). For WW, ADG, and KR, the highest values were recorded in 2020-born individuals, while the lowest were observed in 2019-born lambs, with significant differences among the three years (p<0.05). Among birth months, lambs born in April displayed the highest BW (4.58±0.79 kg), which was significantly greater than that of lambs born in most other months (p<0.05). Lambs born in December achieved the highest WW (33.08±4.10 kg) and ADG (0.32±0.05 kg/d), whereas those born in July showed the lowest performance across all traits, with significant variations between different months (p<0.05). Comparisons across herds revealed that Herds 1, 5, and 9 had higher BW, with Herd 4 showing the lowest BW (3.41±0.68 kg). Herds 1, 2, and 5 performed better in terms of WW and ADG, while Herd 7 exhibited the lowest values. Significant differences were observed for most traits between herds (p<0.05).

### Estimation of variance components for pre-weaning growth traits in Dorper sheep

The variance components and genetic parameters of BW, WW, ADG, and KR estimated by the six models are listed in Table 3. The heritability of BW estimated by different models ranged from 0.0215 (model 5) to 0.0484 (model 4). The maternal heritability ranged from 3.58×10^−7^ (model 5) to 0.0134 (model 4), and the variance ratio of the maternal permanent environmental effects was 0.2880 (model 2) to 0.2941 (model 6). The direct genetic correlation with maternal heritability was −0.9997 for model 4 and −0.9839 for model 6, showing a negatively correlated.

It was estimated that the correlation of WW heritability is between 0.0026 (model 6) and 0.0444 (model 4), maternal heritability is between 2.91×10^−8^ (model 5) and 0.0248 (model 4), the variance ratio of the maternal permanent environmental effects is be-tween 0.3185 (model 6) and 0.3214 (model 2), and that of direct genetic effect with ma-ternal heritability is −0.4121 (model 4) and −0.2054 (model 6).

The ADG heritability estimated by different models was between 0.0391 (model 2) and 0.1295 (model 6), the maternal heritability range was between 8.48×10^−6^ (model 5) and 0.0505 (model 4), and the variance ratio of the maternal permanent environmental effects was between 0.3090 (model 2) and 0.3219 (model 6). There was a highly negative correlation between the direct additive effect and maternal genetic correlation, which was estimated to be −0.8347 by model 6 and −0.7925 by model 6.

The KR heritability estimated by different models was between 0.0501 (model 5) and 0.1489 (model 6), the maternal heritability range was between 0.0003 (model 5) and 0.0726 (model 4), and the variance ratio of the maternal permanent environmental effects was between 0.2730 (model 5) and 0.2980 (model 6). The direct additive effect had a highly negative correlation with the maternal genetic effect, ranging from −0.9785 (model 6) to approximately −0.9556 (model 4).

### Comparison of different animal models

#### Comparison of different models using Akaike information criterion and Bayesian information criterion

The −2LogL, AIC and BIC values for different animal models are listed in Table 4. The AIC and BIC values of model 4 were the largest in terms of BW, WW, ADG and KR. The AIC values were 157.4228, 7,088.7874, −10,614.5899, and −24,305.9189, while the BIC values were 179.8244, 7,111.1609, −10,592.2164, and −24,283.5454, respectively. According to the judgment criteria of AIC and BIC, the smaller the AIC or BIC value, the better the model fit and the more moderate the complexity. The minimum values of AIC for the pre-weaning growth traits (BW, WW, ADG, KR) were 130.4091, 7,056.4486, −10,644.0815, −24,327.6934, respectively. Model 2 (including direct additive genetic and maternal permanent environment effect) was the optimal model for each trait. Based on the BIC analysis, model 2 also provided the best genetic parameter estimates for each trait.

#### Comparison of different models using likelihood ratio test

The LRT results of pre-weaning growth traits are shown in Table 5. There were significant differences among the models regarding BW, WW, ADG, and KR traits. Increasing the maternal permanent environment effect in the model significantly improved the model’s goodness of fit, with Model 2 outperforming the other models. The results of the LRT were consistent with the results of the optimal model derived from the AIC and BIC. Based on optimal models, the direct heritability ( $h_{a}^{2}$) was varied from 0.0215 for BW to 0.0504 for KR. The inclusion of maternal permanent environment effect (*c*^2^) in the model had a significant influence on BW WW, ADG, and KR, reduces the $h_{a}^{2}$ estimate, and they explain 28.8%, 32.14%, 30.9% and 27.31% of the phenotypic variation, respectively.

### Heritability, genetic and phenotypic correlation among traits

The heritability, phenotypic and genetic correlation estimates between pre-weaning growth traits of Dorper sheep are represented in Table 6. The heritability of BW, WW, ADG, and KR were estimated to be 0.0215, 0.0287, 0.0391 and 0.0504, respectively, using optimal model (model 2) for each trait, which belonged to low heritability. BW was negatively genetically correlated with WW (−0.7546), ADG (−0.9728), and KR (−0.4991) traits. WW had a positive genetic correlation with ADG (0.9952) and KR (0.9984). WW also had a positive phenotypic correlation with ADG (0.9829) and KR (0.8819). Consistent with the negative genetic correlations between BW and WW/ADG/KR (Table 6), selecting for larger BW alone would hinder improvements in post-natal growth, emphasizing the need to prioritize maternal environmental factors (e.g., recipient milk quality). So indirect selection for ADG and KR could be achieved by selecting for WW, which gained its own faster genetic progression. WW can be used as an efficient selection index for combined genetic improvement of multiple traits.

## DISCUSSION

Dorper sheep are known for their ability to thrive in a wide range of environments, from arid regions to temperate climates, making them a valuable asset for farmers worldwide. The first-generation hybrids of Dorper sheep crossed with local Chinese sheep breeds exhibit faster weight gain rates and significantly improved meat production performance [24]. Estimation of genetic parameters for pre-weaning growth traits (BW, WW, ADG, KR) in Dorper sheep is important for early selection of breeding.

### Environmental effects

The phenotypic characteristics of growth traits in sheep are shaped by a combination of genetic and environmental factors, including birth year, month, sex, herd, and maternal age. In Dorper sheep, recipient dam age exerted a highly significant effect (p<0.01) on BW, WW, ADG, and KR. This suggests that maternal physiological conditions during gestation and lactation directly influence early postnatal growth traits [25]. Herd effects significantly impacted all pre-weaning traits (p<0.01), highlighting the critical role of standardized management across breeding units. Variations in feeding regimens, health protocols, and housing conditions drove phenotypic divergence, a pattern consistent with findings in Chinese superfine merino sheep [26]. Temporal factors such as birth year and month also played pivotal roles, reflecting seasonal fluctuations in climate and management strategies. Similar temporal effects were observed in Mandya and Corriedale sheep, where birth year strongly influenced BW and WW [27,28]. Sex differences were pronounced in Dorper lambs, with males outperforming females across all traits, aligning with hormonal and metabolic ad-vantages reported in Avikalin sheep [29]. This contrasts with Sonadi sheep, where neither sex nor birth type affected KR [30], suggesting genetic or environmental inter-actions may mitigate sex-based growth disparities in certain breeds. Additionally, while Magotra [31] identified sex and birth type as critical drivers of BW, WW, and ADG across breeds, KR was solely influenced by sex, further emphasizing trait-specific sensitivity to environmental factors.

### Model comparisons

On the basis of environmental effects, we performed genetic parameters and compared six different animal models. The LRT revealed significant differences among the six animal models for all investigated traits (BW, WW, ADG, KR), with Model 2—incorporating both direct additive genetic effects and maternal permanent environmental effects—consistently emerging as the optimal model. This model demonstrated superior goodness of fit, evidenced by the lowest AIC values of traits (AIC = 130.4091 for BW, 7,056.4486 for WW, −10,644.0815 for ADG, and −24,327.6934 for KR). The maternal permanent environmental effects (e.g., uterine conditions, postnatal care) reduces the $h_{a}^{2}$ estimate, and they explain 28.8%, 32.14%, 30.9% and 27.31% of the phenotypic variation, respectively, aligning with findings in Dumeng sheep [4], where maternal environmental contributions were critical for pre-weaning trait. The results of Kushwaha et al [32] demonstrated that direct heritability estimates for all traits were significantly inflated when maternal permanent environmental effects were omitted from the model. Comparative model analyses revealed that biologically reliable and precise heritability estimates could only be achieved by simultaneously incorporating both direct additive genetic effects and maternal permanent environmental effects into the analytical model. Model 2’s optimal fit and low trait heritability are non-contradictory, reflecting the embryo transfer population’s characteristics. Low direct heritability $h_{d}^{2}=0.0026-0.1489$; Table 3) and total heritability ( $h_{t}^{2}=0.0109-0.0565$; Table 3) were primarily driven by two interrelated factors: The LOPU-IVF-ET technology decoupled the genetic source (donor ewes) from the environmental source (recipient ewes), as recipients exclusively determined fetal uterine development and postnatal lactation supply (Materials and Methods). This decoupling substantially diluted the contribution of direct additive genetic effects to phenotypic variation. Maternal additive genetic effects of donors were weakly expressed (maximum maternal heritability $h_{m}^{2}=0.0726$; Table 3), and negative genetic correlations between direct and maternal additive effects (*r_am_* = −0.2054 to −0.9997; Table 3) further offset total genetic variance. Collectively, Model 2 provides the most accurate genetic parameters for guiding Dorper sheep breeding under LOPU-IVF-ET systems, with key implications: optimizing recipient ewe management (e.g., lactation nutrition regulation) will be critical to improving the efficiency of pre-weaning trait improvement.

### Genetic parameters estimate

The heritability estimates for pre-weaning traits in this study were consistently low, with clear population-specific and technical drivers. The heritability estimates for BW in this study (0.0215) was also similar to findings in Awassi sheep [33], was low heritability. Notably, while Tesema et al [34] reported higher total heritability (0.10 for BW) in Dorper × indigenous sheep, this discrepancy primarily arises from population genetic background and reproductive management differences: their crossbred population inherently harbors greater additive genetic variation due to heterosis, whereas our purebred Dorper population (14 sires, 328 dams; Table 1) has limited genetic diversity. Additionally, Dorper×indigenous sheep relied on natural breeding, where maternal genetic and environmental effects are biologically linked, whereas our use of LOPU-IVF-ET decouples the genetic source (donor ewes) from the environmental source (recipient ewes). In our study, fetal development and postnatal lactation depend entirely on the recipient’s uterine environment and milk supply (Materials and Methods), which dilutes direct additive genetic effects—this explains why our BW heritability (0.0215) is lower than that of South African Dorper sheep (0.07) raised under natural breeding [16]. WW exhibited low heritability (0.0287) in this study, in line with trends in Dumeng sheep [4], but lower than estimates for Alpine Merino sheep [35] and Jordan Awassi sheep [36], possibly due to the smaller population size here (n<3,000 vs. n>5,000 in other studies), as larger populations generally yield more accurate heritability estimates. ADG also exhibited low heritability (0.0391) in this study, lower than that reported for Central Anatolian Merino sheep [37], Mecheri sheep [38], Boer goats [39], and Barbari goats [40]. KR showed low heritability (h^2^ = 0.0504), comparable to Sangsari sheep [14], models that included direct additive genetic effects and maternal permanent environment effects had significant effects on KR. Large values for maternal permanent environmental effects consistent with the biological characteristics of ruminant embryonic development under assisted reproduction. Maternal permanent environmental effects strongly impacted early growth traits, as lambs depend on maternal milk supply pre-weaning and are sensitive to environmental changes (e.g., feeding conditions). Small changes in the feeding environment, nutritional quality of feed, drinking water conditions and feeding management level of the ewes may have a direct or indirect significant effect on the WW of the lambs, preweaning daily weight gain and other growth indicators. Thus, the effect of maternal permanent environment is particularly prominent in this critical period before weaning.

### Genetic and phenotypic correlation

Estimates using the best animal model 2 showed that the absolute values of the genetic correlation coefficients for BW, WW, ADG, and KR ranged from 0.4991 to 0.9984. BW was negatively genetically correlated with WW (−0.7546), ADG (−0.9728), and KR (−0.4991) traits. The study of Behrem [37] stated that the genetic correlation coefficient between newborn weight and WW of Merino sheep was −0.30, and the study of Arzik et al [11] concluded that the genetic correlation between BW and WW was negative, which is the same as the results of the present study. Negative genetic correlations between BW and post-weaning growth traits suggesting that direct selection for BW may have an antagonistic effect on the improvement of later growth traits to avoid compromising overall breeding progress by simply pursuing initial BW. WW had a positive genetic correlation with ADG (0.9952) and KR (0.9984). WW also had a positive phenotypic correlation with ADG (0.9829) and KR (0.8819). The high genetic correlation between ADG, WW and KR is similar to that expressed by Similar results to Ghafouri-Kesbi et al [41] estimate of 0.91 for Mehraban sheep and Gholizadeh and Ghafouri-Kesbi [42] estimate of 0.93 for Baluchi sheep, and Shokrollahi and Baneh [43] in Arabi sheep. So indirect selection for ADG and KR could be achieved by selecting for WW, which gained its own faster genetic progression. WW can be used as an efficient selection index for combined genetic improvement of multiple traits.

## CONCLUSION

Model 2 was optimal model for analyzing BW, WW, ADG, KR. These traits showed low heritability (0.0215–0.0504), indicating challenges in direct genetic improvement. Strong trait relationships were observed, with genetic correlation coefficients between WW and ADG, WW and KR, ADG and KR were 0.9952, 0.9984, and 0.9597, respectively. While corresponding phenotypic correlations were 0.9829, 0.8819, and 0.9437. The heritability of early growth traits is at a low level and genetic selection for improvement is relatively slow. Genetic improvement of other growth traits can be achieved by selection for WW. Breeders should prioritize recipient dam nutrition (e.g., energy intake during lactation) to enhance WW, leveraging its high genetic correlation with ADG and KR to accelerate genetic gain under intensive management.

## Figures and Tables

**Table 1:** Description statistics for early growth traits of Dorper sheep

Item	BW (kg)	WW (kg)	ADG (kg/d)	KR
No. of animals in pedigree	2,022	2,022	2,022	2,022
No. of sires	14	14	14	14
No. of dams	328	323	323	323
No. of records	1,999	1,985	1,985	1,985
Average number of progenies per sire	142.78	141.78	141.78	141.78
Average number of progenies per dam	6.09	6.15	6.15	6.15
Mean	3.94	29.12	0.28	0.02
SD	0.72	4.41	0.05	0.001
CV (%)	18.38	15.14	17.44	6.69

No. of animals in pedigree is the total number of animals; No. of sires is the quantity of male parents in the pedigree; No. of dams is the quantity of female parents in the pedigree; No. of records is the total number of data records for each trait; Mean is the arithmetic mean value of the trait data.

BW, birth weight; WW, weaning weight; ADG, average daily gain weight from birth to weaning; KR, Kleber ratio from birth to weaning (ADG/WW^0.75^); SD, standard deviation; CV, coefficient of variation.

**Table 2:** Least squares mean (±SD) for pre-weaning traits of Dorper sheep

Factors	n	BW (kg)	n	WW (kg)	n	ADG (kg/d)	n	KR
Overall	1,999	3.94±0.72	1,985	29.12±4.41	1,985	0.28±0.05	1,985	0.02±0.001
Dam age^1)^		^**^		^**^		^**^		^**^
2	326	4.03±0.68^a^	326	27.46±3.41^c^	326	0.26±0.04^c^	326	0.022±0.001^c^
3	687	3.80±0.80^b^	681	30.01±4.80^a^	681	0.29±0.05^a^	681	0.023±0.002^a^
4	986	4.01±0.67^a^	978	29.07±4.25^b^	978	0.28±0.05^b^	978	0.022±0.001^b^
Sex		^**^		^**^		^**^		^**^
Male	1,091	4.01±0.72^a^	1,085	30.68±4.41^a^	1,085	0.30±0.05^a^	1,085	0.023±0.001^a^
Female	908	3.86±0.72^b^	900	27.24±3.60^b^	900	0.26±0.04^b^	900	0.022±0.001^b^
Birth year		^**^		^**^		^**^		^**^
2019	129	4.08±0.77^a^	129	26.77±3.00^c^	129	0.25±0.03^c^	129	0.021±0.001^c^
2020	609	3.64±0.69^b^	606	30.12±4.61^a^	606	0.29±0.05^a^	606	0.023±0.001^a^
2021	1,261	4.07±0.69^a^	1,250	28.88±4.31^b^	1,250	0.28±0.05^b^	1,250	0.022±0.001^b^
Birth month		^**^		^**^		^**^		^**^
2	22	4.22±1.05^bc^	22	32.07±2.98^a^	22	0.31±0.03^ab^	22	0.023±0.001^ab^
3	275	4.01±0.46^bcd^	273	27.76±3.56^c^	273	0.26±0.04^d^	273	0.022±0.001^c^
4	124	4.58±0.79^a^	122	29.17±4.86^bc^	122	0.27±0.05^cd^	122	0.022±0.001^c^
5	86	4.29±0.96^ab^	85	28.69±4.30^bc^	85	0.27±0.05^cd^	85	0.022±0.002^c^
7	6	3.80±1.19^d^	6	22.72±3.42^d^	6	0.21±0.03^e^	6	0.020±0.001^d^
8	427	3.69±0.89^d^	426	28.76±4.69^bc^	426	0.28±0.05^cd^	426	0.022±0.002^bc^
9	236	3.71±0.55^d^	234	29.93±4.26^b^	234	0.29±0.04^bc^	234	0.023±0.001^b^
10	198	4.00±0.61^bcd^	198	27.90±3.58^bc^	198	0.27±0.04^d^	198	0.022±0.001^c^
11	494	3.97±0.62^bcd^	490	29.28±4.21^bc^	490	0.28±0.05^cd^	490	0.022±0.001^bc^
12	131	3.94±0.40^cd^	129	33.08±4.10^a^	129	0.32±0.05^a^	129	0.023±0.001^a^
Herd		^**^		^**^		^**^		^**^
1	13	4.52±0.94^a^	13	33.06±3.00^a^	13	0.32±0.04^a^	13	0.023±0.001^a^
2	7	3.83±1.25^b^	7	31.54±1.92^ab^	7	0.31±0.02^ab^	7	0.023±0.001^a^
3	494	4.02±0.58^b^	493	28.41±3.51^c^	493	0.27±0.04^d^	493	0.022±0.001^b^
4	374	3.41±0.68^c^	372	29.87±4.10^bc^	372	0.29±0.05^bc^	372	0.023±0.001^a^
5	126	3.99±0.32^b^	124	33.27±3.99^a^	124	0.33±0.04^a^	124	0.023±0.001^a^
6	179	3.83±0.77^b^	179	28.88±4.90^c^	179	0.28±0.05^cd^	179	0.022±0.002^b^
7	83	4.07±0.70^b^	83	25.45±3.39^d^	83	0.24±0.03^e^	83	0.021±0.001^c^
8	126	3.92±0.43^b^	124	27.91±3.48^c^	124	0.27±0.04^d^	124	0.022±0.001^b^
9	74	4.70±0.87^a^	72	31.22±4.71^ab^	72	0.29±0.05^bc^	72	0.022±0.001^b^
10	523	4.13±0.72^b^	518	28.82±4.69^c^	518	0.27±0.05^cd^	518	0.022±0.002^b^

1) Age of recipient Dam.

a–e Within a column, means without a common superscript letter differ significantly, p<0.01.

** p<0.01.

SD, standard deviation; BW, birth weight; WW, weaning weight; ADG, average daily gain; KR, Kleiber ratio.

**Table 3:** Estimates of the (co)variance components and the genetic parameters for pre-weaning growth traits

Traits	Models	$\sigma_{a}^{2}$	$\sigma_{m}^{2}$	*σ* *_am_*	$\sigma_{c}^{2}$	$\sigma_{e}^{2}$	$\sigma_{p}^{2}$	$h_{d}^{2}$	$h_{m}^{2}$	*c* ^2^	$h_{t}^{2}$	*r* *_am_*
BW	Model 1	0.0097				0.3699	0.3796	0.0256			0.0256	
	Model 2	0.0082			0.1093	0.2619	0.3794	0.0215		0.2880	0.0215	
	Model 3	0.0097	5.29×10^−7^			0.3699	0.3796	0.0256	1.39×10^−6^		0.0256	
	Model 4	0.0179	4.97×10^−4^	−0.0095		0.3674	0.3715	0.0484	0.0134		0.0109	−0.9997
	Model 5	0.0082	1.36×10^−7^		0.1093	0.2619	0.3794	0.0215	3.58×10^−7^	0.2880	0.0215	
	Model 6	0.0158	0.0041	−0.0079	0.1096	0.2589	0.3726	0.0424	0.0110	0.2941	0.0109	−0.9839
WW	Model 1	0.5179				12.5291	13.0469	0.0397			0.0397	
	Model 2	0.3738			4.1894	8.4727	13.0358	0.0287		0.3214	0.0287	
	Model 3	0.3641	0.0694			12.5866	13.0201	0.0280	0.0053		0.0333	
	Model 4	0.5731	0.3209	−0.1767		12.3811	12.9217	0.0444	0.0248		0.0418	−0.4121
	Model 5	0.3723	3.78×10^−7^		4.1895	8.4738	13.0355	0.0286	2.91×10^−8^	0.3214	0.0286	
	Model 6	0.0332	0.2458	−0.0185	4.1398	8.6164	12.9981	0.0026	0.0189	0.3185	0.0186	−0.2054
ADG	Model 1	7.56×10^−5^				0.0015	0.0016	0.0482			0.0482	
	Model 2	6.13×10^−5^			4.84×10^−4^	0.0010	0.0016	0.0391		0.3090	0.0391	
	Model 3	7.54×10^−5^	2.62×10^−8^			0.0015	0.0016	0.0481	1.67×10^−5^		0.0481	
	Model 4	1.83×10^−4^	7.59×10^−5^	−9.35×10^−5^		0.0014	0.0015	0.1220	0.0505		0.0481	−0.7925
	Model 5	6.13×10^−5^	1.33×10^−8^		4.85×10^−4^	0.0010	0.0016	0.0391	8.48×10^−6^	0.3090	0.0391	
	Model 6	1.95×10^−4^	7.19×10^−5^	−9.87×10^−5^	4.83×10^−4^	0.0009	0.0015	0.1295	0.0479	0.3219	0.0459	−0.8347
KR	Model 1	8.25×10^−8^				1.38×10^−6^	1.46×10^−6^	0.0565			0.0565	
	Model 2	7.37×10^−8^			3.99×10^−7^	9.89×10^−7^	1.46×10^−6^	0.0504		0.2731	0.0504	
	Model 3	8.17×10^−8^	6.84×10^−10^			1.38×10^−6^	1.46×10^−6^	0.0560	0.0005		0.0565	
	Model 4	1.97×10^−7^	9.82×10^−8^	−1.33×10^−7^		1.32×10^−6^	1.35×10^−6^	0.1458	0.0726		0.0218	−0.9556
	Model 5	7.32×10^−8^	5.08×10^−10^		3.99×10^−7^	9.89×10^−7^	1.46×10^−6^	0.0501	0.0003	0.2730	0.0504	
	Model 6	2.02×10^−7^	9.70×10^−8^	−1.37×10^−7^	4.03×10^−7^	9.25×10^−7^	1.35×10^−6^	0.1489	0.0716	0.2980	0.0184	−0.9785

$\sigma_{a}^{2}$, direct additive genetic variance; $\sigma_{m}^{2}$, maternal additive genetic variance; *σ_am_*, direct-maternal genetic covariance; $\sigma_{c}^{2}$, maternal permanent environmental variance; $\sigma_{e}^{2}$, residual variance; $\sigma_{p}^{2}$, phenotypic variance; $h_{d}^{2}$,direct heritability; $h_{m}^{2}$, maternal heritability; *c*^2^, variance ratio of the maternal permanent environmental effects; $h_{t}^{2}$, total heritability; *r_am_*, direct-maternal genetic correlation; BW, birth weight; WW, weaning weight; ADG, average daily gain; KR, Kleiber ratio.

**Table 4:** Standard values of AIC and BIC information for traits in different animal models

Traits	Models	−2 log *L*	AIC	BIC
BW	Model 1	150.3102	154.3102	165.5110
	Model 2	124.4091	130.4091	147.2103
	Model 3	150.3104	156.3104	173.1116
	Model 4	149.4228	157.4228	179.8244
	Model 5	124.4092	132.4092	154.8108
	Model 6	123.6413	133.3472	161.3492
WW	Model 1	7,080.3235	7,084.3235	7,095.5102
	Model 2	7,050.4486	7,056.4486	7,073.2287
	Model 3	7,080.2461	7,086.2461	7,103.0263
	Model 4	7,080.7874	7,088.7874	7,111.1609
	Model 5	7,050.4484	7,058.4484	7,080.8219
	Model 6	7,052.0188	7,061.7003	7,089.6671
ADG	Model 1	−10,623.4113	−10,619.4113	−10,608.2246
	Model 2	−10,650.0815	−10,644.0815	−10,627.3014
	Model 3	−10,623.4111	−10,617.4111	−10,600.6311
	Model 4	−10,622.5899	−10,614.5899	−10,592.2164
	Model 5	−10,650.0810	−10,642.0810	−10,619.7075
	Model 6	−10,649.0980	−10,639.4199	−10,611.4530
KR	Model 1	−24,314.2694	−24,310.2694	−24,299.0826
	Model 2	−24,333.6934	−24,327.6934	−24,310.9133
	Model 3	−24,314.2471	−24,308.2471	−24,291.4669
	Model 4	−24,313.9189	−24,305.9189	−24,283.5454
	Model 5	−24,333.6632	−24,325.6632	−24,303.2897
	Model 6	−24,333.5532	−24,323.8512	−24,295.8843

−2 log *L*, −2×likelihood ratio test; AIC, Akaike information criterion; BIC, Bayesian information criterion; BW, birth weight; WW, weaning weight; ADG, average daily gain; KR, Kleiber ratio.

**Table 5:** Likelihood ratio test results of pre-weaning growth traits

Model comparison	DF	BW	WW	ADG	KR
Model 2/Model 1	1	25.9011^***^	29.8749^***^	26.6702^***^	19.4240^***^
Model 3/Model 1	1	−0.0002^ns^	0.0774^ns^	−0.0002^ns^	−0.0223^ns^
Model 4/Model 1	2	0.8874^ns^	−0.4639^ns^	−0.8214^ns^	−0.3505^ns^
Model 5/Model 1	2	25.9010^***^	29.8751^***^	26.6697^***^	19.3938^***^
Model 6/Model 1	3	26.6689^***^	28.3047^***^	25.6867^***^	19.2838^***^
Model 5/Model 2	1	−0.0001^ns^	0.0002^ns^	−0.0005^ns^	−0.0302^ns^
Model 6/Model 2	2	0.7678^ns^	−1.5702^ns^	−0.9835^ns^	−0.1402^ns^
Model 4/Model 3	1	0.8876^ns^	−0.5413^ns^	−0.8212^ns^	−0.3282^ns^
Model 5/Model 3	1	25.9012^***^	29.7977^***^	26.6699^***^	19.4161^***^
Model 6/Model 3	2	26.6691^***^	28.2273^***^	25.6969^***^	19.3061^***^
Model 6/Model 4	1	25.7815^***^	28.7686^***^	26.5081^***^	19.6343^***^
Model 6/Model 5	1	0.7679^*^	−1.5704^*^	−0.9830^*^	−0.1100^*^

ns, non-significant (p>0.05);

* p<0.05,

*** p<0.001.

DF, degree of freedom; BW, birth weight; WW, weaning weight; ADG, average daily gain; KR, Kleiber ratio.

**Table 6:** Genetic and phenotypic correlation for pre-weaning early growth traits in Dorper sheep

Trait	BW	WW	ADG	KR
BW	0.0215^a^	0.1631	−0.0073	−0.2987
WW	−0.7546	0.0287^a^	0.9829	0.8819
ADG	−0.9728	0.9952	0.0391^a^	0.9437
KR	−0.4991	0.9984	0.9597	0.0504^a^

The heritability in the table is indicated by the superscript ^a^ along the diagonal.

The genetic correlation is below the diagonal, and the phenotypic correlation is above the diagonal.

BW, birth weight; WW, weaning weight; ADG, average daily gain; KR, Kleiber ratio.

## Data Availability

Upon reasonable request, the datasets of this study can be available from the corresponding author.
